# Pneumorrhachis: An uncommon radiological entity

**DOI:** 10.4102/sajr.v25i1.2255

**Published:** 2021-11-29

**Authors:** Atish Vanmali, Kamlesh D. Daji

**Affiliations:** 1 Department of Diagnostic Radiology, Jackpersad and Partners, Durban, South Africa

**Keywords:** pnuemorrhachis, spontaneous pneumomediastinum, epidural air

## Abstract

Pneumorrhachis (PR) is a rare and interesting phenomenon, in which air is present within the spinal canal. The aetiologies are varied, broadly grouped as traumatic, non-traumatic or iatrogenic. Pneumorrhachis secondary to spontaneous pneumomediastinum (SPM) and barotrauma of the lungs is uncommon and even rarer within the paediatric group. This report describes a paediatric patient presenting with a persistent cough who developed a SPM and subsequent PR.

## Introduction

Pneumorrhachis (PR) is a rare clinical entity in which air is present within the extradural or intradural compartments of the spinal canal.^1^ It is rarely associated with pneumomediastinum, particularly in young children.^2^ The common causes listed include epidural trauma from spinal fracture, epidural instrumentation for lumbar puncture, epidural anaesthesia or ‘spontaneous pneumomediastinum’.^1^ Case reports have described PR in the setting of direct trauma to the spine or skull and within the context of pneumomediastinum, pneumopericadium and pneumoperitoneum.^3^ Awareness of the benign nature of the evolution of PR in the presence of pneumomediastinum is important to prevent additional imaging, instrumentation and other unnecessary measures.^3^

## Case report

An 8-year-old female presented to the family medicine specialist with a 2-day history of persistent cough, fever and dyspnoea. Her past medical history was insignificant, with no history of asthma or prior hospital admissions. Her immunisation complied with the National Immunisation Schedule.

**FIGURE 1:**
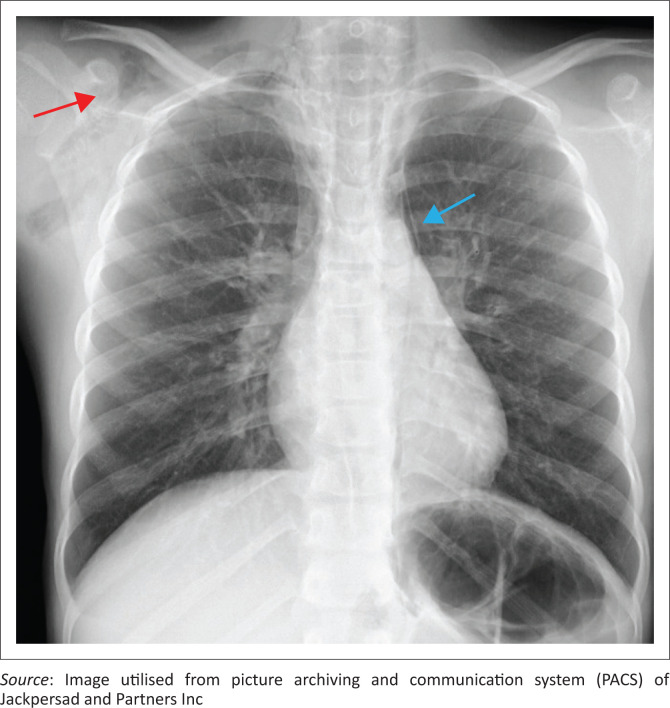
Chest radiograph on admission demonstrating subcutaneous emphysema (red arrow) and pneumomediastinum (blue arrow).

On admission, her oxygen saturation, measured by pulse oximetry (SpO_2_) was normal (99% – 100%). A chest radiograph (Figure 1) demonstrated pneumomediastinum and subcutaneous emphysema.

**FIGURE 2:**
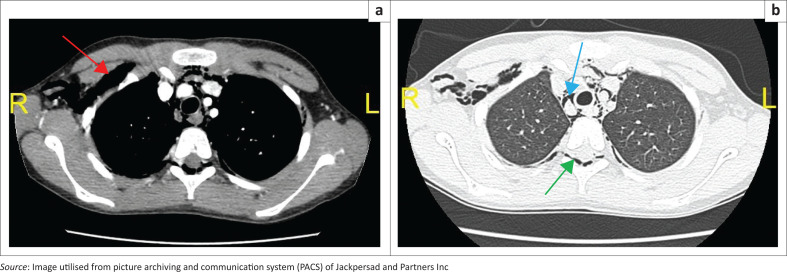
(a and b) Axial post contrast (CT) scan, soft tissue and lung settings, respectively, demonstrating subcutaneous emphysema (red arrow), pneumomediastinum (blue arrow) and epidural emphysema (green arrow).

For improved delineation of the extent of the spontaneous pneumomediastinum (SPM), an urgent CT scan of the chest (Figure 2a and 2b) was performed. Extensive pneumomediastinum and subcutaneous emphysema was demonstrated.

**FIGURE 3:**
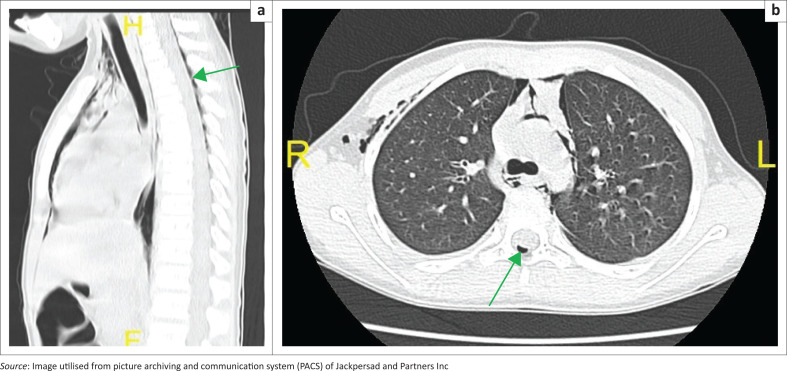
(a and b) Sagittal reconstructed image through the thoracic spine and axial image at level of carina, lung settings, demonstrating epidural emphysema (green arrow).

Air tracking into the posterior epidural space (pneumorrhachis) at multiple levels of the cervical and thoracic spinal canal was also observed (Figure 3a and b). Bilateral lower lobe bronchial wall thickening was noted but there were no features of atelectasis or air trapping.

**FIGURE 4:**
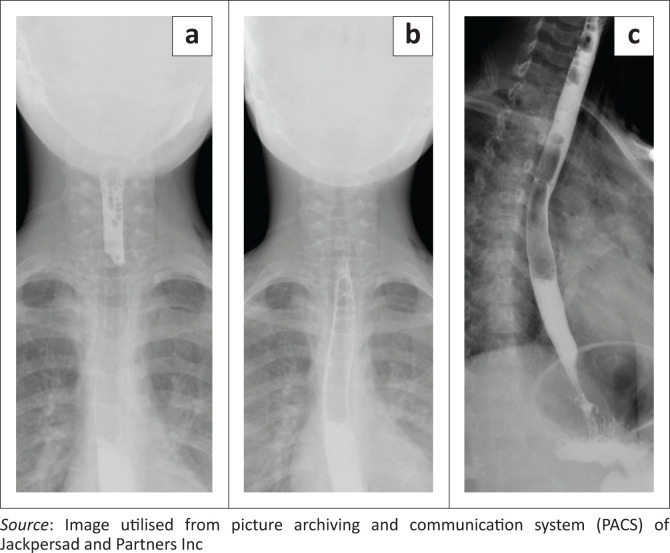
(a, b and c) Contrast swallow of the cervical and thoracic oesophagus excluded oesophageal perforation.

A water-soluble contrast swallow excluded any oesophageal perforation (Figure 4). The patient remained comfortable and was managed conservatively with medical treatment demonstrating improvement of symptoms. The patient was subsequently discharged and follow up was scheduled in three weeks.

## Discussion

Pneumomediastinum is a benign entity, defined as free air within the mediastinum and classified as spontaneous or secondary.^4^ Secondary pneumomediastinum that requires urgent intervention is secondary to recent intervention, history of aero-digestive organ injury, cervico-thoracic infection, pneumothorax or mechanical ventilator-related injury. Spontaneous pneumomediastinum is diagnosed without any cause.^4^ Although SPM is associated with a benign clinical course, an important and difficult differential to exclude is oesophageal perforation or Boerhaave’s syndrome. Oesophageal perforation that arises from spontaneous rupture is associated with higher mortality than the iatrogenic or traumatic causes.^5^

Spontaneous pneumomediastinum and subcutaneous emphysema (Hamman syndrome) results from rupture of the alveoli secondary to increased intrabronchial pressure that may occur in the background of coughing, shouting, vomiting or after labour.^6^ The Macklin effect refers to the increase in alveolar pressure that results in alveolar rupture. The alveolar air dissects through the pulmonary interstitium along the bronchovascular sheaths towards the hila and mediastinum.^7^

The most common symptoms of SPM are dyspnoea, cough, neck pain, chest pain and dysphagia. Subcutaneous emphysema is the commonest clinical finding followed by Hamman’s sign, which is pathognomonic of pneumomediastinum.^8^ Hamman’s sign refers to the crunching sound over the mediastinum, synchronous with the heartbeat.^8^

A rare complication of SPM is PR, which was demonstrated in the case presented. Air within the cervical subarachnoid space producing a ‘pneumomyelogram’ was initially described by Gordon et al.^9^ This was documented on the background of trauma and subsequently described under various terms. The term PR was first applied 10 years later by Newbold et al.^10^

Pneumorrhachis is anatomically classified into intradural (intraspinal air within the subdural or subarachnoid space) and extradural (intraspinal, epidural air). Intradural PR is associated with major trauma and suggestive of severe injury, and extradural PR is usually innocuous.^11^

The aetiology of PR may be classified into traumatic, non-traumatic and iatrogenic. Air can enter the spinal canal from traumatic spinal leaks or penetrating injuries. Iatrogenic causes are related to recent surgical or anaesthetic manipulations. Non-traumatic causes occur as a result of respiratory complications secondary to high intrathoracic pressure and barotrauma.^11^ The other non-traumatic aetiologies are associated with degenerative, malignant, inflammatory and infectious disease by a gas forming organism.^12^ Pneumorrhachis in the context of trauma indicates severe injury.^8^

**FIGURE 5:**
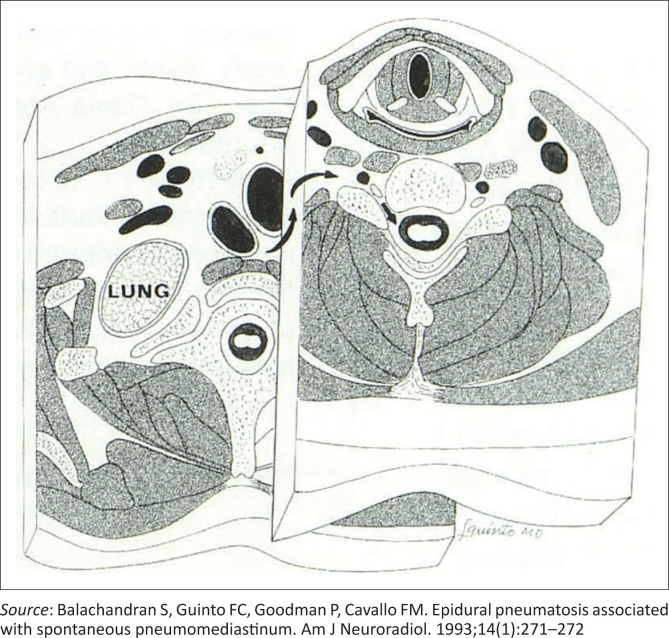
Diagram of the lower cervical and upper thoracic cross sections illustrating the pathway of air dissection (arrows) leading to epidural pneumatosis.

In 1989, Tsuji et al. described the first CT demonstration of spinal epidural emphysema following bronchial asthma and violent coughing.^13^ Pneumorrhachis secondary to SPM occurs as a result of communication of the posterior mediastinum and the epidural space and the absence of a fascial barrier.^8^ Air typically accumulates along the posterior epidural space because of its lower resistance compared with the anterior vascular network.^8^ In 1993 Balachandran et al. hypothesised that the absence of fascial barriers between the posterior mediastinum and the epidural space facilitated free mediastinal air to dissect into the paraspinal tissues and subsequently travel through the neural foramina alongside the neurovascular bundle into the epidural space (Figure 5).^14^

Epidural air is usually benign, however PR related neurological symptoms have been documented.^3^ Yaginuma et al. presented two cases of PR associated with pneumomediastinum in young patients.^2^ Their literature search of children less than 15 years documented 25 articles reporting 32 cases, in which PR associated with pneumomediastinum occurred in all cases.^2^

The investigations of choice for a patient with PR include plain radiographs and CT.^11^ In the setting of a traumatic aetiology, radiographs are essential to detect possible associated injuries. A linear lucency projected over the spinal canal on a lateral chest radiograph is suggestive of PR. CT is the gold standard for reliable detection of PR and its wider application in the assessment of adjacent anatomy in a non-traumatic or traumatic cause.^11^

Following assessment of the primary cause, treatment is primarily directed to the aetiology. This patient presented with SPM and PR. Upon exclusion of an oesophageal perforation and history of trauma, she was managed conservatively. The expectation was that the air would reabsorb over 2 to 3 weeks and the administration of a high concentration of inspiratory oxygen would promote the absorption of air.

## Conclusion

In the absence of trauma, aero digestive organ injury and iatrogenic causes, PR represents a rare benign association of pneumomediastinum that commonly occurs secondary to barotrauma of the lung and the Macklin effect. Understanding the mechanism in which air reaches the epidural space allows a conservative approach to treatment without unnecessary investigations. Neurological symptoms are rare and most patients experience reabsorption of the epidural air over two to three weeks without long-term complications, emphasising the innocuous course of this phenomenon.
